# Walking on sunshine! Vitamin D in psychiatric inpatients

**DOI:** 10.1192/bjo.2021.235

**Published:** 2021-06-18

**Authors:** Emma Davies, Lucy Mountford

**Affiliations:** Surrey and Borders NHS Foundation Trust

## Abstract

**Aims:**

We aimed to determine whether vitamin D is being tested on admission for psychiatric inpatients at a local inpatient hospital, to identify the level of vitamin D for this group and to establish whether vitamin D treatment provided is according to NICE guidance.

Emerging evidence suggests that psychiatric patients are more vulnerable to vitamin D deficiency, due to reduced sun exposure, social isolation, long inpatient stays and poor diet. Low vitamin D levels may also increase susceptibility to SARS CoV-2 infection and COVID-19 severity.

**Method:**

Standards were determined by local policies, RCPsych recommendations and NICE guidance. Data were collected retrospectively from electronic patient records and entered manually to a spreadsheet for analysis.

**Result:**

67% of patients had vitamin D tested on admission to hospital. Of the patients that had their vitamin D level tested, 39% patients had their result recorded. 48% either had a low vitamin D level or required replacement. 6 of 12 patients with a documented low vitamin D level had the correct vitamin D treatment, according to NICE guidance.

**Conclusion:**

Of 46 patient records, nearly half had a documented low vitamin D level or were on treatment. We would therefore suggest that vitamin D testing should form part of the routine admission bloods. It is an important opportunity to detect deficiency or insufficiency for a potentially vulnerable group of patients. Intervention is simple and effective.

Results demonstrated room for improvement for vitamin D testing on admission to hospital, thus improving potential treatment and benefits for individual patients. The importance of recording blood results on to the electronic patient record was also highlighted.

We raised awareness and provided further education to all junior doctors, with creative posters and informative communications. Following the implementation of these changes a re-audit of 40 patients showed 75% had vitamin D tested on admission or during and of these, 58% either had a low vitamin D level or required replacement. 7 of 9 patients with a documented low vitamin D level had the correct vitamin D treatment, according to NICE guidance. Within this closed loop audit, we have reported moderate improvement in the testing of vitamin D for patients on admission to hospital along with a significant improvement in the treatment of vitamin D deficiency, according to NICE guidance.

